# Transient Global Amnesia as the Sole Presentation of an Acute Stroke in the Left Cingulate Gyrus

**DOI:** 10.1155/2019/4810629

**Published:** 2019-02-20

**Authors:** Lawrence Chau, Antonio Liu

**Affiliations:** Department of Neurology, Adventist Health White Memorial, USA

## Abstract

We discuss a patient who presented to our hospital with signs, symptoms, and clinical course consistent with transient global amnesia (TGA). Her hospital work-up was overall unremarkable except for the presence of a diffusion weighted imaging (DWI) abnormality on MRI suggestive of acute infarction in the left cingulate gyrus. Symptoms quickly resolved, and she was discharged home in stable condition. While the etiology of TGA remains controversial, in discussing this case and the current literature we hope to provide further data supporting the possibility of an underlying ischemic process, as well as help better illustrate the neurologic structures involved.

## 1. Introduction

Transient global amnesia is a clinical phenomenon described as the sudden onset of acute amnesia (mostly anterograde, but retrograde is also frequently observed) with neither altered consciousness nor cognitive impairment. It subsequently resolves within 24 hours, and there are no known long-term sequelae aside from a mild amnestic period in some patients. In the decades since it was first observed, its characteristics have been defined clearly, yet the cause remains elusive. Vascular processes, epilepsies, and migraines have been postulated as possible etiologies, yet the lack of prevalence of these disorders in cases of TGA as noted in prior studies suggests against them as causes [1]. Another hypothesis is that TGA may be associated with ischemia, and while the evidence is sparse, multiple case reports have been written to explore this theory. The hippocampus has typically been sited as the affected structure [2, 3], as review of MRIs in retrospective cases of TGA has shown a predominance of DWI hyperintensities in that region [4, 5]. Here, we report a case of TGA in downtown Los Angeles with findings in the left cingulate gyrus as seen on DWI, a much rarer occurrence. In doing so, we hope to provide more data to the medical community regarding both incidence and areas of involvement in ischemic TGA to better our understanding of this condition.

## 2. Case

**Figure 1:**
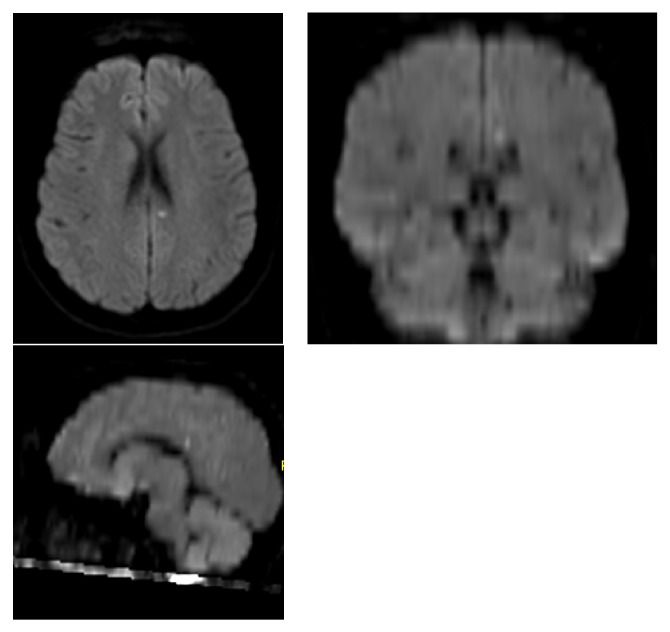
DWI in axial, coronal, and sagittal views (Siemens Aera 1.5T) (ST 5mm, TE 16ms, TR 5230ms) showing a single diffusion abnormality in left cingulate gyrus.

**Figure 2:**
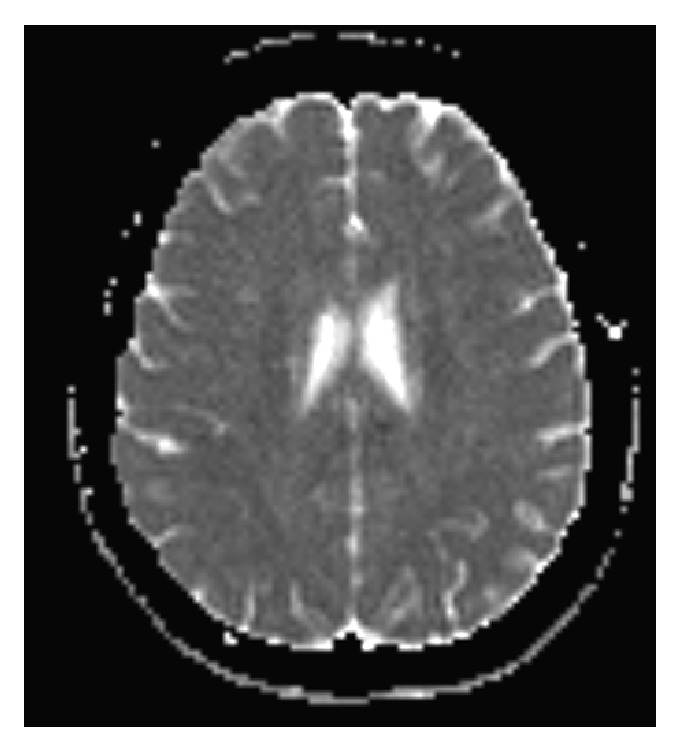
ADC view (same values).

A 60-year-old woman was initially brought to the emergency room by concerned family members. Her only past medical history is obesity, hyperlipidemia, and hyperthyroidism, for which she takes levothyroxine 75mcg and simvastatin 40mg. She also takes a daily aspirin 325mg. Family described that the patient had a sudden change in her behavior just prior to admission. There was no reported loss of consciousness or altered level of consciousness, just noting that she was acting “strange.” She was cooking at the time and suddenly left the food unattended on the stove. She appeared lost and seemed to have forgotten what she was doing. She remained generally oriented with no focal complaints, and there was no headache or pain. She could walk, and she had no weakness of any kind. There was no precipitating seizure and no incontinence. However, she was not following conversations and did not recognize her own sister who dropped by for dinner. En route to the hospital as well as in the emergency room, she kept asking the very same question, “where am I?” and “how did I get here?” Someone will offer the answer only to be met with the same questions minutes later. During her ER stay, she had a completely normal and non-focal neurologic examination. NIHSS score was zero. She scored 3/3 on registration but was unable to recall objects after a while. She had no idea how she got to the emergency room, but she is oriented to person and time. Her toxicology screen was negative. Blood pressure was 117/71 and patient was afebrile throughout her hospital course. Further work-up for altered mental status was unremarkable, chemistry and CBC were negative, GFR 113, and random sugar was 97. TSH was 5.96. There was no evidence of acute infection. Initial CT of the head was negative. She was admitted to the hospital for overnight observation. The next morning, patient was back to her baseline but still had no idea how she got to the hospital. The last event she recalled from the day prior was leaving the bathroom and walking towards her living room, apparently before she started cooking. She knew she had a complete lapse of memory and was concerned about developing dementia like her father. Her registration and recall returned to normal. Family at bedside also confirmed that she appeared to be at her baseline. Her repeat neurological examination that morning remained non-focal. MRI of the brain, however, showed a small DWI signal abnormality at the left cingulate gyrus (see Figures 1 and 2). No other lesions were noted. Stroke workup eventually showed no other abnormality and she was sent home on Plavix and Lipitor 80mg daily.

## 3. Discussion

Currently, cases of transient global amnesia with DWI findings has typically implicated the hippocampus. Naldi et al. reported a clear case of TGA in 2016 with findings of DWI hyperintensity in the right hippocampus on MRI and stenosis of the right posterior cerebral artery on CT angiography. Even then, they further cited multiple prior cases with similar hippocampal involvement [2], albeit small variations in clinical presentation, such as in Carota et al. where the patient had additional findings of anomic aphasia (coined a “TGA plus” syndrome) [3]. In a retrospective study of 86 patients with TGA, Enzinger et al. found that, of the 10 patients with significant DWI findings, there was a predominance of lesions in the hippocampus [5]. Forster et al. in Germany looked at 386 patients diagnosed with TGA at their hospital, and, of the 27 patients who received MRI evaluation, all 12 with DWI lesions were in the hippocampus [4]. Furthermore, it was noted that the properties of these lesions compared to those of pure hippocampal strokes were indistinguishable in terms of DWI and apparent diffusion coefficient (ADC) [6], which seems to suggest that these are true infarctions.

TGA with findings solely in the cingulate gyrus, as was seen with our patient, is a significantly rare occurrence. To our understanding, only one other case has been reported. Gallardo-Tur et al. In 2014 described a patient who presented with classical TGA symptoms with no recurrence of symptoms noted on a one-year follow-up. MRI findings at the time 3 days after presentation is significant for findings in the right cingulate gyrus. In our patient, clinical presentation was similar, with the exception that imaging findings were noted on the left. Other case reports of TGA with DWI hyperintensities include involvement of the corpus callosum [7, 8], putamen [9], uncinate gyrus [10], and the fornix [11]. Thus, while the affected neurologic structures that have been reported in TGA are all in some manner associated with memory or mental development, the fact that a similar clinical presentation arises from various affected structures only highlights how the current literature is lacking in data to establish a comprehensive relationship between ischemia and transient amnesia.

Also, it should be reiterated that despite the numerous cases of TGA with DWI lesions reported, the overall incidence remains low. As noted by Enzinger et al. and Forster et al., only 11% and 3% of all TGA cases seen at their institutions, respectively, were positive for significant MRI findings. This is likely in part because TGA remains a clinical diagnosis, and decision to obtain further work-up with MRI and work-up for ischemia is to the discretion of the provider. Thus, the true incidence of ischemic events in TGA may be underreported. Detection methods and timing of MRI studies may also be limiting factors, as Scheel et al. in a retrospective study of 198 TGA cases showed a significant increase in detection of lesions when slice thickness of 3 mm is utilized and when MRI studies are performed 2 days after the amnestic event [12]. Furthermore, in the retrospective study by Enzinger et al., the hyperintensity findings drastically differed amongst patients with similar presentations, as 9 lesions were found in the left hemisphere, 5 were in the right, and 2 were found bilaterally. Lesion sizes varied between 3 and 6 mm. Comparatively, patients with pure hippocampal strokes could have no memory involvement whatsoever [6]. Thus, the fact that neither the presence, nor the location, nor the size of DWI hyperintensities can consistently correlate with the severity of TGA findings only highlights how lacking the current literature is in understanding the significance of these ischemic-like lesions in the pathogenesis of TGA. An ischemic event may only be a factor in a cascade of neurologic processes that lead to an amnestic event, possibly alongside concurrent metabolic or vascular processes that have also been implicated as possible causes [1] and as such requires further study. Thus, while this is not the first documented case of TGA with ischemic findings in the cingulate gyrus, it takes a step forward in expanding the literature's currently limited understanding of the involvement of ischemic events in TGA. Hopefully, this will help us as the scientific community better map the structures other than the hippocampus that are involved. Doing so may provide a better correlation between structural involvement and disease, furthering the possibility of ischemia or other etiology of cerebrovascular involvement as the culprit of this clinical condition and aid in refining the diagnostic criteria.
